# The LacI family protein GlyR3 co-regulates the *celC* operon and *manB* in *Clostridium thermocellum*

**DOI:** 10.1186/s13068-017-0849-2

**Published:** 2017-06-24

**Authors:** Jinlyung Choi, Dawn M. Klingeman, Steven D. Brown, Chris D. Cox

**Affiliations:** 1 Department of Chemical and Biomolecular Engineering, University of Tennessee, Knoxville, TN 37996 USA; 2 Center for Environmental Biotechnology, University of Tennessee, Knoxville, TN 37996 USA; 3 BioEnergy Science Center, Oak Ridge National Laboratory, Oak Ridge, TN 37831 USA; 4 Biosciences Division, Oak Ridge National Laboratory, Oak Ridge, TN 37831 USA; 5 Department Civil and Environmental Engineering, University of Tennessee, Knoxville, TN 37996 USA

**Keywords:** *Clostridium thermocellum*, GlyR3, LacI, CcpA, *manB*

## Abstract

**Background:**

*Clostridium thermocellum* utilizes a wide variety of free and cellulosomal cellulases and accessory enzymes to hydrolyze polysaccharides present in complex substrates. To date only a few studies have unveiled the details by which the expression of these cellulases are regulated. Recent studies have described the auto regulation of the *celC* operon and determined that the *celC*–*glyR3*–*licA* gene cluster and nearby *manB*–*celT* gene cluster are co-transcribed as polycistronic mRNA.

**Results:**

In this paper, we demonstrate that the GlyR3 protein mediates the regulation of *manB.* We first identify putative GlyR3 binding sites within or just upstream of the coding regions of *manB* and *celT*. Using an electrophoretic mobility shift assay (EMSA), we determined that a higher concentration of GlyR3 is required to effectively bind to the putative *manB* site in comparison to the *celC* site. Neither the putative *celT* site nor random DNA significantly binds GlyR3. While laminaribiose interfered with GlyR3 binding to the *celC* binding site, binding to the *manB* site was unaffected. In the presence of laminaribiose, in vivo transcription of the *celC*–*glyR3*–*licA* gene cluster increases, while *manB* expression is repressed, compared to in the absence of laminaribiose, consistent with the results from the EMSA. An in vitro transcription assay demonstrated that GlyR3 and laminaribiose interactions were responsible for the observed patters of in vivo transcription.

**Conclusions:**

Together these results reveal a mechanism by which *manB* is expressed at low concentrations of GlyR3 but repressed at high concentrations. In this way, *C. thermocellum* is able to co-regulate both the *celC* and *manB* gene clusters in response to the availability of β-1,3-polysaccharides in its environment.

**Electronic supplementary material:**

The online version of this article (doi:10.1186/s13068-017-0849-2) contains supplementary material, which is available to authorized users.

## Background

*Clostridium thermocellum* is an anaerobic, thermophilic, Gram-positive bacterium that has a highly efficient cellulolytic system [1]. This bacterium is considered a model organism for biofuels processing since it combines cellulolytic and ethanologenic abilities [1–5]. Cellulolytic activity is conferred by a combination of free glycoside hydrolases and an extracellular multi-enzyme cellulase complex called the cellulosome [6–12]. *C. thermocellum* ATCC 27405 is the reference strain. Strains YS and AD2 were used in many of the key studies which developed the cellulosome concept and have recently been sequenced [13]. An efficient transformation methodology has been developed for strain DSM 1313 [14] facilitating the development of an engineered strain capable of high ethanol titter [15].

*Clostridium thermocellum* employs more than 100 genes for biomass degradation, including more than 70 genes that encode for various cellulosomal enzymes [16]. The cellulosome has a core, scaffold protein called CipA that binds to the surface of the bacterial cell, to the catalytic subunits, and to the carbohydrate-binding module (CBM) [17]. Various CBM and catalytic subunits may be deployed to provide cellulolytic activity specific to various biomass substrates [18]. While many studies have described the structural and catalytic activity of the cellulosome and free cellulases, relatively few investigations have focused on the regulation of these genes; the most significant of these are reviewed below.

Recently it was determined that many cellulosomal genes are regulated by a common mechanism involving the σ^I^ alternative transcription factor, which binds to the core RNA polymerase to form a holoenzyme capable of transcribing these genes [19]. In the absence of polysaccharides, SigI is inactivated via binding to the anti-sigma factor N-terminal domain of the trans-membrane protein RsgI. The conformation of RsgI changes upon binding of a target extracellular polysaccharide to the C-terminal CBM of the RsgI protein, thereby releasing SigI to the cytoplasm of the cell and up regulating SigI-regulated genes, including *sigI* and many cellulosomal genes. Various SigI–RsgI proteins are activated by specific polysaccharides, thereby providing specificity in regulation of cellulosomal genes [20].

In contrast, the *celC* operon, containing the *celC*, *glyR3*, and *licA* genes, is regulated by a different mechanism involving the LacI family protein GlyR3, which negatively auto-regulates the operon by binding to the *celC* promoter region to repress its expression [16]. The repression of the operon is relieved in the presence of laminaribiose, which interferes with GlyR3 binding to the promoter. Regulation of the *celC* operon is perhaps the most well characterized of the non-cellulosomal cellulases in *C. thermocellum*. CelC is a non-cellulosomal endoglucanase affiliated to the glycoside hydrolase family 5, which is one of the largest of the glycoside hydrolase families. LicA is an endo-1,3-β-d-glucosidase. Recently it has been shown that the nearby *manB*–*celT* gene cluster is co-regulated with *celC* by an unknown mechanism [17]. It was also shown that the *manB*–*celT* gene cluster was transcribed as a single operon [17]. ManB is a cellulosomal family 26 glycoside hydrolase and CelT is a cellulosomal family 9 endoglucanase [21, 22]. A recent paper [23] created *glyR1*, *glyR2*, and *glyR3* knock-out strains to demonstrate that LacI proteins in *C. thermocellum* controlled expression of specific hemicellulases.

In this paper, we identify a new site within the coding region of *manB* to which GlyR3 binds. We demonstrate an inverse relationship between *glyR3* and *manB* gene expression and show that the interactions between GlyR3 and laminaribiose are responsible for this expression pattern. We extend the current regulatory model of the *celC* operon in *C. thermocellum* to include a GlyR3-dependent mechanism by which *manB* is regulated. In other Gram-positive organisms, LacI family proteins similar to GlyR3 are known to repress numerous carbon metabolism pathways [24]. This result opens the possibility of GlyR3 playing a larger role in regulating *C. thermocellum* cellulolytic activity than previously known.

## Results

### Protein and DNA sequences suggest similarities in DNA binding between GlyR3 in *C. thermocellum* and CcpA in *B. subtilis*

**Fig. 1:**
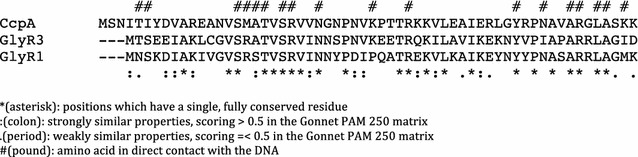
Basis for using CcpA binding sites in *B. subtilis* to identify GlyR3 binding sites in *C. thermocellum*. Amino acid sequence alignment of the first 60 amino acid residuals of CcpA in *B. subtilis* and GlyR1 and GlyR3 in *C. thermocellum* using clustal omega v1.2.0 [37]. A total of 11 and 14 of the 18 amino acid residuals in GlyR1 and Glyr3, respectively, in direct contact with the DNA are identical to those in CcpA [38]

CcpA is a global regulatory protein in *Bacillus subtilis* that is known to regulate at least 44 different operons [25]. CcpA binds with phosphorylated HPr in the presence of glucose and subsequently suppresses catabolic pathways for other sugars by binding with the *cre* control sequences of their catabolic genes [26]. Although the regulatory domains and mode of action of CcpA and LacI family proteins are completely different, CcpA has a helix-turn-helix DNA binding domain similar to many LacI family proteins. We aligned the helix-turn-helix domains of CcpA and the GlyR1, GlyR2 and GlyR3 LacI family proteins in *C. thermocellum* to determine the similarity of their DNA binding domains. Two of the LacI family proteins, GlyR1 and GlyR3, show a high degree of similarity over their first 60 residues (Fig. 1). Of the 18 amino acid residues in CcpA in direct contact with DNA, there were 11 and 14 exact matches for GlyR1 and GlyR3, respectively. We also observed that the GlyR3 binding site (TGAACGCGCGTACA) in the *celC* operon was similar to the consensus CcpA binding site in *B. subtilis* (TGNAANCGNWNNCW). These two observations led us to hypothesize that CcpA could be used as a model to identify additional GlyR3 binding sites in *C. thermocellum.*

### Putative GlyR3 binding sites associated with *manB* and *celT* are identified

The DNA sequences of the 44 known CcpA binding sites in *B. subtilis* [25] were used to construct a position-specific scoring matrix (PSSM) (Additional file 1: Table S1). The performance of the PSSM in identifying CcpA binding sites in *B. subtilis* is given by the receiver operating characteristic (ROC) curve (Additional file 1: Figure S1), which is a graphical illustration of the performance of a threshold in a binary classifier system [27]. The ROC curve demonstrates that with a threshold value of 12 the PSSM is able to identify a high fraction of CcpA sites in *B. subtilis* (high sensitivity) while maintaining a relatively low false positive rate (1-specificity). Experiments are required to discriminate true positives from false positives.

Based on the similarity in sequences of CcpA and GlyR3 binding sites, we used the *B. subtilis*-derived PSSM to search for additional potential GlyR3 binding sites in *C. thermocellum.* The PSSM provided information about the relative importance of each base within the sequence that would not have been available had we searched using the *celC* GlyR3 binding-site sequence alone. The PSSM score of the GlyR3 binding site of *celC* is 9.18 demonstrating that the PSSM derived from CcpA may be useful in identifying potential GlyR3 binding sites in *C. thermocellum.* Putative GlyR3 binding sites near the *celC* operon were identified by scanning the *C. thermocellum* genome using the PSSM for CcpA and are listed in Table 1. A putative GlyR3 binding site for *manB* with a PSSM score of 14.62 was identified. Since the score of the *manB* site was greater than the score for *celC* (9.18), this was a promising site for GlyR3 binding. In addition, a putative GlyR3 binding site for *celT* was also identified. While the *celT* PSSM score of 5.78 was significantly lower than that of *celC* and *manB*, it was significantly greater than the average PSSM score for a random location in the overall genome (−14.08).

**Table 1:** Sequences and PSSM informational scores (bits) of putative GlyR3 binding sites

Sequences	Absolute position	Genes	PSSM score (bits)	Number of palindrome positions
TGAAAGC|GCTTTCA^a^		Optimum sequence based on *B. subtlis*	31.92	7
TGAACGC|GCGTACA^b^	3308248..3308261	celC (Cthe_2807)	9.18	6
TGTAAAC|GGTGTCA	3317291..3317304	manB (Cthe_2811)	14.62	4
GTAAATC|GGTTGCA	3318880..3318893	celT (Cthe_2812)	5.78	3
Average over the *C. thermocellum* genome			−14.08	–

^a^Determined by analysis of identified CcpA binding sequences in Ref. [25]

^b^Sequence from Ref. [16]

### GlyR3 binds to the putative binding site in the *manB* coding region

**Fig. 2:**
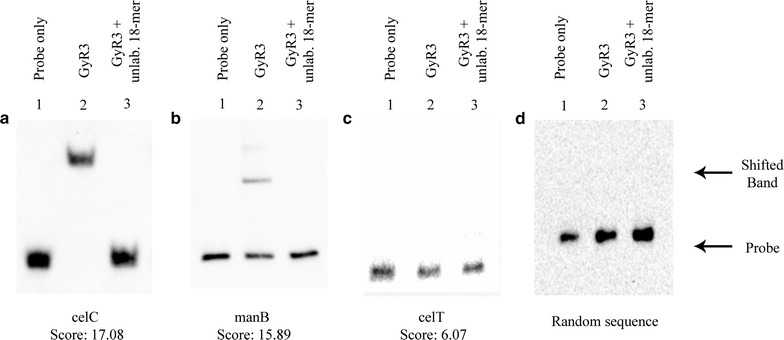
Protein–DNA interactions at potential GlyR3 binding sites near *celC*, *manB*, and *celT* genes. Electrophoretic Mobility Shift Assay was used to assess binding of GlyR3 to putative DNA binding sites for **a** *celC*, **b** *manB*, **c** *celT*, and **d** a random sequence of DNA. Figure shows GlyR3 binding to the DNA sites near *celC* and *manB* but no binding to the site near *celT* or to random DNA. **a** *Lane 1 celC*; *Lane 2 celC* + GlyR3; *Lane 3 celC* + GlyR3 + unlabeled 18-mer. **b** *Lane 1 manB*; *Lane 2 manB* + GlyR3; *Lane 3 manB* + GlyR3 + unlabeled 18-mer. **c** *Lane 1 celT*; *Lane 2 celT* + GlyR3; *Lane 3 celT* + GlyR3 + unlabeled 18-mer. **d** *Lane 1* random DNA; *Lane 2* DNA + GlyR3; *Lane 3* DNA + GlyR3 + unlabeled 18-mer. In all cases, the target DNA is 0.2 ng (*celC* = 0.156 nM, *manB* = 0.187 nM, *celT* = 0.196 nM, control = 0.204 nM), GlyR3 is 150 ng (387 nM), and 18-mer competitive DNA is 500 ng (2.14 mM)

Electrophoretic mobility shift assays (EMSA) were used to further investigate protein–DNA interactions in the presence and absence of laminaribiose for the GlyR3 binding regions of *celC*, *manB*, and *celT*. The binding region *celC* was included to show consistency with the binding behavior previously reported in Ref. [16]. In the absence of laminaribiose, addition of GlyR3 results in a strong shift in the *celC* band, a partial shift in the location of the *manB* band, and no shifting of the *celT* or random DNA bands (Fig. 2).

The observed faint top band in lane 2 of Fig. 2b can be attributed to high-molecular-weight DNA–GlyR3 aggregates [28]. The GlyR3-induced shifts of the *celC* and *manB* bands were reversed upon addition of competitor DNA (unlabeled 18-mer), confirming that the putative binding sites were responsible for the GlyR3 binding. Control experiments using GlyR1 confirmed that it was not able to bind to the GlyR3 binding sites of either the *celC* or *manB*, despite similarities in the DNA binding domains of the two LacI proteins (Additional file 1: Figure S2).

**Fig. 3:**
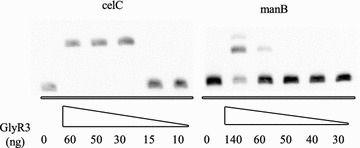
Relative strength of *celC* and *manB* GlyR3 binding stites. EMSA of *celC* and *manB* binding sites as a function of GlyR3 level. The assay reveals that GlyR3 binds more readily to the binding site in *celC* compared to the binding site in *manB*. DNA loading was 0.2 ng (*celC*: 0.156 nM, *manB*: 0.187 nM). GlyR3 loadings were: 140 ng (361 nM), 60 ng (155 nM), 50 ng (129 nM), 40 ng (103 nM), 30 ng (77 nM), 15 ng (39 nM), and 10 ng (26 nM)

**Fig. 4:**
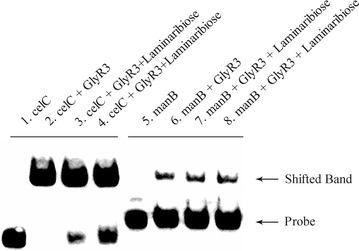
Effect of laminaribiose on interaction of GlyR3 with DNA. EMSA was used to determine the effect of laminaribiose addition on GlyR3 binding to DNA binding sites. Laminaribiose disrupted the binding of GlyR3 to the *celC* binding site but had no measurable effect on interactions between GlyR3 and the *manB* binding site. *Lane 1 celC* [0.2 ng (0.156 nM)]; *Lane 2 celC* + GlyR3 [150 ng (387 nM)]; *Lane 3 celC* + GlyR3 + laminaribiose [35 μg (50 mM)]; *Lane 4 celC* + GlyR3 + laminaribiose [70 μg (100 mM)]; *Lane 5 manB* [0.2 ng (0.187 nM)]; *Lane 6 manB* + GlyR3[150 ng (387 nM)]; *Lane 7 manB* + GlyR3 + laminaribiose [35 μg (50 mM)]; *Lane 8 manB* + GlyR3 + laminaribiose [70 μg (100 mM)]

The strength of DNA binding by GlyR3 was further investigated using a titration test in which the GlyR3 concentration was increased while keeping the DNA concentration constant (Fig. 3). It was observed that GlyR3 interactions with the *celC* binding domain were insignificant at levels less than or equal to 15 ng of protein but that all DNA was bound by GlyR3 at levels equal to or greater than 30 ng. In contrast, levels equal to or greater than 60 ng of GlyR3 were needed to achieve significant binding of the *manB* binding domain. The band was partially shifted upon addition of 60 ng and further shifted at 140 ng of GlyR3. Overall the data in Fig. 3 suggest that the *celC* binding domain has a higher affinity for GlyR3 than the *manB* binding domain. Addition of 35 and 70 μg of laminaribiose was shown to relieve GlyR3 repression in a dose-dependent manner (lanes 2, 3, 4 are shifted 99, 82, 67%, respectively, as calculated by ImageJ), consistent with previous reports [16] (Fig. 4). In contrast, GlyR3 binding to the *manB* binding site appeared to be unaffected by laminaribiose addition (lanes 6, 7, 8 are shifted 18, 18, 19%, respectively, as calculated by ImageJ). Therefore, the effect of laminaribiose on GlyR3 binding to DNA was different for the two binding sites, which may affect the way in which laminaribiose controls expression of the regulated genes.

### In vivo expression of *manB* is repressed in the presence of laminaribiose

**Fig. 5:**
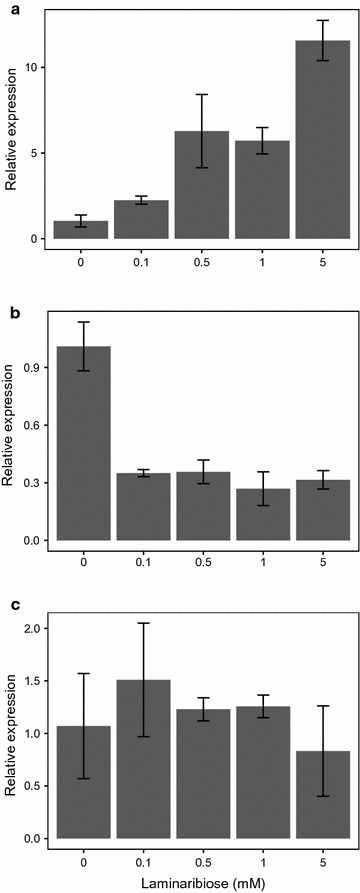
In vivo expression of *celC*, *manB*, and *celT* via qRT-PCR. Relative gene expression in *C. thermocellum* as a function of laminaribiose concentration as determined by quantitative RT-PCR. **a** *celC*; **b** *manB*; **c** *celT*. The results show that laminaribiose increases *celC* expression, decreases *manB* expression, and has no effect on *celT*

We determined the effect of adding laminaribiose on the expression of *celC, manB*, and *celT* in *C. thermocellum* using qRT-PCR. *C. thermocellum* was incubated at 60 °C for 1 h after adding different concentrations of laminaribiose to each anaerobic serum bottle. The data were normalized to the housekeeping gene *recA*. Gene expression changes were determined by the comparative CT method using a control sample to which no laminaribiose was added [29]. As shown in Fig. 5a, transcription of *celC* increased with laminaribiose concentration. All samples showed an increase in gene expression compared to samples without laminaribiose. In contrast, *manB* was repressed by low quantities of laminaribiose (<0.1 mM) (Fig. 5b). Expression of *celT* was unaffected by laminaribiose addition (Fig. 5c).

**Fig. 6:**
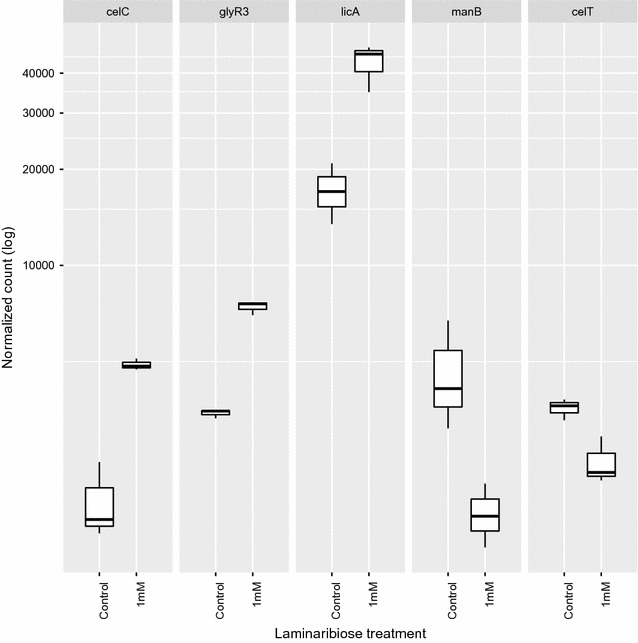
In vivo expression of *celC*, *glyR3, licA, manB*, and *celT* via RNA-seq. Effect of 1 mM laminaribiose on expression of genes in the *celC* and *manB* gene clusters

We also confirmed the in vivo expression determined by qRT-PCR with RNA Seq. *Clostridium thermocellum* was grown at 60 °C in MTC medium with Avicel. Laminaribiose (1 mM) was added at late-exponential phase then cells were harvested 1 h later. The RNA Seq data (Fig. 6) show a similar pattern as Fig. 5; addition of 1 mM laminaribiose results in an increase in expression in *celC*, *glyR3,* and *licA,* and a decrease in expression of *manB.* In addition, the RNA-Seq results show a slight decrease in expression of *celT,* in contrast to qRT-PCR data that showed the expression of *celT* to be unaffected by laminaribiose addition. RNA-seq is more sensitive than qRT-PCR, which may explain this minor difference in results.

### The role of GlyR3 in repressing *manB* expression is confirmed by in vitro transcription assay

**Fig. 7:**
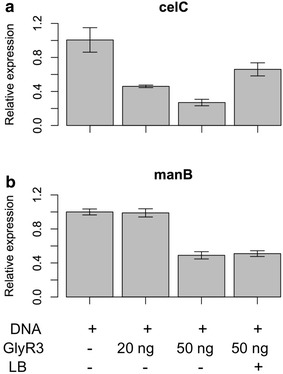
In vitro transcription assay. In vitro relative gene expression as a function of laminaribiose concentration as determined by quantitative RT-PCR. **a** *celC* (DNA: 2.65 nM, GlyR3: 20 ng (51.6 nM), 50 ng (128.9 nM), laminaribiose: 50 mM); **b** *manB* (DNA: 2.05 nM, GlyR3: 20 ng (51.6 nM), 50 ng (128.9 nM), laminaribiose: 50 mM)

An in vitro transcription assay using a DNA template containing only the promoter and coding region of *celC* and *manB* was conducted in order to verify that the interactions between GlyR3 and laminaribiose were responsible for the observed in vivo transcription profiles rather than some unidentified mechanism. As shown in Fig. 7a, expression of *celC* was repressed by exogenously added GlyR3 in a dose-dependent manner (*p* value = 0.00294 for 20 ng GlyR3 vs. DNA only and *p* value = 0.0013 for 50 ng vs. 20 ng GlyR3). Upon addition of laminaribiose repression by exogenous GlyR3 was relieved (*p* value = 0.00141 between GlyR3 50 ng and GlyR3 50 ng + laminaribiose). This result showed a same pattern as observed by Newcomb et al. [16]. Figure 7b shows that *manB* was not repressed upon addition of 20 ng of GlyR3 (*p* = 0.74) but was repressed at 50 ng of GlyR3 (*p* = 0.0001). However, the repression of *manB* was not affected by laminaribiose (*p* = 0.64) at a dosage of 50 ng of GlyR3. This pattern is consistent with the results of the EMSA and in vivo expression assays.

## Discussion

**Fig. 8:**
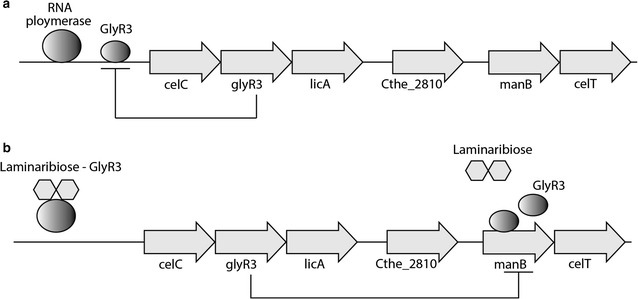
Model of GlyR3 regulation. Expanded model of *celC* regulon. **a** In the absence of laminaribiose, GlyR3 auto suppresses the expression of the *celC* operon, resulting in relatively low GlyR3 concentrations, thereby allowing expression of *manB*. **b** In the presence of laminaribiose, repression of the *celC* operon is relieved resulting in high GlyR3 concentrations and repression of *manB*

Our results suggest an extended model of the *celC* regulon that includes regulation of *manB* via GlyR3 (Fig. 8). In the model proposed by Newcomb et al., expression of the *celC* operon is autorepressed in the absence of laminaribiose due to binding of GlyR3 to the *celC* promoter [16], but the mechanism by which *manB* is regulated is not defined. We have identified a site in the *manB* coding region to which GlyR3 binds. Our EMSA results show that binding of GlyR3 to the *manB* binding site is relatively weak compared to GlyR3 binding to the *celC* binding site. Therefore, the low concentration of GlyR3 that is likely to occur when the *celC* operon is autorepressed (yet still somewhat leaky) may be insufficient to repress the expression of *manB*. Indeed, *manB* expression was observed in the absence of laminaribiose in the gene expression results presented here (Figs. 5b, 6, 7) and earlier by Newcomb et al. [17].

Laminaribiose relieves the repression of GlyR3 according to the *celC* model of Newcomb [16]. Under these conditions, GlyR3 may be expressed at sufficiently high levels to bind to the *manB* binding site, thereby blocking expression of *manB*. Gene expression data presented (Figs. 5b, 6, 7) confirm that *manB* expression decreases in the presence of laminaribiose (Fig. 5b). Consistent with this observation, Newcomb [17] also observed a decrease in *manB* expression in *C. thermocellum* when it is grown on laminarin in comparison to cellulose. In contrast to the effect of laminaribiose on GlyR3 binding to the *celC* binding site, GlyR3 binding to the *manB* binding site is mostly unaffected by laminaribiose according to EMSA experiments (Fig. 4).

Overall, this model allows for repression of *manB* and expression of the *celC* operon to be under control of laminaribiose. This contrary behavior is critically dependent both upon the weaker relative binding of GlyR3 to the *manB* binding site and the apparent lack of effect of laminaribiose on the GlyR3–*manB*-binding-site complex. The mechanism by which laminaribiose would decrease binding at the *celC* site, but not affect binding at the *manB* site is unknown.

Our model of the extended *celC* regulon is consistent with recent gene expression studies using LacI knock-out strains of *C. thermocellum* DSM 1313 [23].We compared the expression levels of *celC*, *glyR3*, *licA*, *manB*, and *celT* for the wild type, ΔglyR1 and ΔglyR3 strains during mid-exponential, late-exponential, and stationary growth phase by analyzing data from this study (Additional file 1: Figure S3). The expression levels for wild type and ΔglyR1 strains were indistinguishable from one another for all genes and growth phases, indicating that GlyR1 does not regulate these genes. In comparison to the wild type, the ΔglyR3 strain showed increase expression of *celC*, and *licA* in the absence of the GlyR3 repressor for all growth phases. Expression levels of *manB* and *celT* were similar for the wild type and ΔglyR3 strains. This observation is consistent with our model in which physiologically-relevant concentrations of GlyR3 in the wildtype in the absence of laminaribiose are too low to affect *manB* or *celT* expression.

Our ESMA results showed no evidence of GlyR3 binding to the potential GlyR3 binding site near the celT gene (Fig. 2c). Newcomb [17] presented evidence that *manB* and *celT* form an operon which produces polycistronic mRNA when transcribed. Our gene expression data showed that while *manB* expression was regulated by laminaribiose, *celT* expression was unaffected (Fig. 5c) or decreased in concert with *manB* expression (Fig. 6). Cumulatively, this evidence demonstrates that the potential GlyR3 binding site near *celT* was not functional.

The cellulosome of *C. thermocellum* has unusually high activity on crystalline cellulose, allowing it to access sugars in recalcitrant substrates within its ecological niche. In addition to cellulose, plant biomass contains a significant fraction of hemicellulose. To access to cellulose, *C. thermocellum* deploys a number of enzymes with activity toward the spectrum of β-1,4 and β-1,3 linkages present in biomass hemicellulose. In the presence of laminaribiose, a β-1,3 disaccharide, GlyR3 downregulates genes encoding for two cellulosomal hemicellulases (*manB* and *celT*) while upregulating two genes that encode for non-cellulosomal hemicellulases (*celC* and *licA*). One possible explanation for this observation is that *C. thermocellum* uses laminaribiose to sense the presence of hemicellulosic activity within its local environment, either its own or the activity of another organism. In the presence of significant local hemicellulosic activity, catalytic activity of the cellulosome may be shifted away from hemicellulosic activity toward cellulosic activity by downregulating *manB* and *celT*. These speculations are limited by the fact that our study focuses on the regulation of a handful of cellulase among dozens expressed and under conditions greatly simplified compared to natural conditions. Global gene expression studies under various environmental conditions are beginning to appear in the literature [23, 30, 31]; however, an understanding of the details of cellulase regulation remains elusive and will require additional studies.

## Conclusions

We identified a site within the coding region of the *manB* gene that binds GlyR3. The binding affinity of this site to GlyR3 appears to be weaker than the previously identified GlyR3 binding site near the transcriptional start site of *celC*. Laminaribiose appears to have little effect on binding GlyR3 to the *manB* binding site, in contrast to the antagonistic effect laminaribiose appears to have between GlyR3 binding to the *celC* site. In vivo expression of *manB* was greatest in the absence of laminaribiose and was repressed in the presence of laminaribiose. These results were consistent with an in vitro transcription assay which showed that *manB* expression was greatest at low GlyR3 concentrations and that addition of laminaribiose did not reverse the repression caused by high concentrations of GlyR3. Together, these results suggest an extended model for GlyR3-mediated regulation of the *celC*–*manB* gene cluster and reveal the potential for complex regulatory mechanisms of polysaccharide-active genes that are dependent upon the available substrates in the environment.

## Methods

### Bioinformatics analysis

The DBTBS transcriptional regulation database (http://dbtbs.hgc.jp) [25] was used to identify 44 CcpA binding sites in *B. subtilis*, and a consensus sequence was determined. The frequency of each base at each of the 14 positions were normalized to determine the information content of the sequence [32], and the position specific scoring matrix (PSSM) was determined. The receiver operating characteristic (ROC) curve of this operator was determined by calculating the PSSM score over each 14mer in the *B. subtilis* genome and calculating the true positive and false positive rates for each PSSM score threshold between 9 and 19. This matrix was used to search for possible GlyR3 binding sites in the proximity of the *manB*–*celT* gene cluster in *C. thermocellum*.

### Bacterial strains

Bacterial strains and plasmids used in this study are summarized in Additional file 1: Table S2. The *glyR3* gene was cloned into the pTXB1 (New England Biolabs) expression vector and transformed into *E. coli* Top10 (Invitrogen). The plasmids were harvested and purified by Minipreparation kit (Wizard^®^ Plus Minipreps, Promega) and transformed into T7 Express Competent *E. coli* strain C2566 (New England Biolabs) for production of GlyR3 (Additional file 1: Table S2).

### Culture conditions

*Clostridium thermocellum* cultures were prepared under anaerobic conditions in 100 ml batch serum bottles and grown at 60 °C in chemically defined (Medium for Thermophilic Clostridia) MTC medium prepared as described by Zhang et al. [33]. Avicel (PH105, FMC Biopolyer, Philadelphia, PA) was used as the carbon source. *E. coli* were grown in liquid culture with shaking at 37 °C in Luria–Bertani (LB) medium containing 100 μg/ml ampicillin. Expression of *glyR3* was induced with 0.5 mM isopropyl thiogalactoside (IPTG) when an OD_600_ of 0.4 was obtained. *E. coli* colonies for screening and selection were grown on LB medium agar with 100 μg/ml ampicillin at 37 °C.

### Cloning of *glyR3*

Genomic DNA was extracted from *C. thermocellum* using the Wizard^®^ Genomic DNA Purification Kit (Promega) and was used as a template for the amplification of the *glyR3* gene. The target DNA was PCR amplified using PuReTaq™ Ready-To-Go™ PCR beads (GE Health care) following Ref. [16]. *Eco*RV and *Xho*I were used for restriction sites [16] at 37 °C overnight after washing by MinElute PCR purification kit (Qiagen) (Additional file 1: Table S3, primers 1 and 2). The target DNA was inserted into the NruI and XhoI sites of the pTBX1 plasmid (New England Biolabs) and transformed into *E. coli* (Oneshot Top10, Invitrogen). The colonies were selected on an ampicillin (100 μg/ml) plate. The target plasmid was extracted using a Minipreparation kit (Wizard^®^ Plus Minipreps, Promega) and then sequenced for verification.

### Expression and purification of GlyR3

GlyR3 was obtained using an expression vector and purified following the procedures of Ref. [16]. The cloned vector was transformed into T7 Express Competent *E. coli* (Additional file 1: Table S2) and induced with 0.5 mM IPTG. Expression of the target protein was verified using SDS-PAGE. GlyR3 was purified following the IMPACT system protocol (New England Biolabs). The purified GlyR3 concentration was measured using the Bradford (Bio-rad) method with bovine serum albumin as a standard.

### Electrophoretic mobility shift assay (EMSA)

DNA fragments from *celC*, *manB* and *celT* were amplified with biotin labeled primers 3–10 (Additional file 1: Table S3). GlyR3 protein was obtained as described above. Running buffer was prepared following the LightShift Chemiluminescent EMSA kit Protocol. Electrophoresis was done under 100 mV for 30 min with TBE (Tris-Bis-EDTA) gel (Invitrogen) which was transferred to nylon paper. The signal was developed using the LightShift Chemiluminescent EMSA kit. The image was detected by ChemiDOC XRS + (Bio-Rad) with 30 s exposure. Unlabeled 18-mers matching the sequence of interest were used to competitively bind GlyR3 and provide confirmation of the binding locations. The effect of laminaribiose (Megazyme) on GlyR3–DNA interactions was also assessed using EMSA.

### RNA extraction

To preserve RNA for qRT-PCR analysis, live cells were centrifuged at 10,000*g* for 5 min. The cells were resuspended in 10 volumes of RNAlater (Qiagen) and incubated for 15 min. The cells were centrifuged again and 1 ml of TRIzol (Invitrogen) was added before freezing at −80 °C. After thawing, the cells were subjected to three 20 s cycles of bead beating (FastPrep-24, MP Biomedical). Chloroform (250 μl) was added to the lysate before vortexing for 45 s. The contents were centrifuged after a 3 min rest and the upper layer was collected and mixed with ethanol (1:1 volume). RNA was purified using an Ultra CleanTM Microbial RNA isolation kit (MO BIO). DNA contamination was removed by addition of DNase I (Qiagen) to the membrane of the kit.

### Quantitative real-time PCR

Brilliant ^®^II SYBR Green QRT-PCR Master Mix kit (Agilent technologies) was used with 10 ng total RNA and 100 nM each of forward and reverse primer as listed in Additional file 1: Table S3 (17–22). New primers were designed using Primer3.

### mRNA Sequencing

Two datasets, SRP074026 and SRP057818 [23], were obtained from NCBI and independently analyzed to verify transcript level of *celC*, *glyR3*, *licA*, *manB,* and *celT*. Raw sequence reads were mapped on to *Clostridium thermocellum* reference sequence (NC_009012 and NC_017304) using Bowtie2 [34]. Gene count was obtained using HTSeq [35]. The final count was normalized using DEseq2 [36].

### In vitro transcription assay

In this assay, the *celC* promoter and coding region and *manB* coding region which was generated using primers 23–26 (Additional file 1: Table S3) was inserted into pCR2.1-TOPO vector. The plasmid vector was transformed into TOP 10 *E. coli* to be selected on ampicillin contained LB plate. The DNA template was generated using primers 24, 26, and 27 (Additional file 1: Table S3) to amplify the promoter and coding region of *celC* and *manB*. TranscriptAid T7 High Yield Transcription Kit (Thermo scientific) was used to perform the in vitro transcription assay. The samples were treated with DNase according to the protocol from manufacturer. The RNA product was isolated using the TRIzol method (Invitogen) along with RNeasy mini kit (Qiagen). The length of transcripts for celC (853 bp) and manB (1467 bp) were confirmed via Bioanalyzer. Quantifying mRNA expression was performed by 1-step SYBR Green QRT-PCR (Agilent Technologies) with the primers 17–20 (Additional file 1: Table S3).

## Additional file

**Additional file 1.** Additional figures and tables.

## Abbreviations

EMSA: electrophoretic mobility shift assay
MTC: medium for thermophilic clostridia
PSSM: position-specific scoring matrix
ROC: receiver operating characteristic
