# Supplementary material for: The LacI family protein GlyR3 co-regulates the celC operon and manB in Clostridium thermocellum
Source: Biotechnol Biofuels. 2017 Jun 24;10:163. doi: 10.1186/s13068-017-0849-2 (PMC5483248; doi:10.1186/s13068-017-0849-2)
Supplement: Supplementary file 1 — Additional file 1. Additional figures and tables. [file 13068_2017_849_MOESM1_ESM.pdf]

Additional File 1.

Figures

Figure S1. Receiver Operator Curve for PSSM scored CcpA binding sites in *B. subtilis*.

Figure S2. EMSA controls for GlyR1 binding to *celC* and *manB* GlyR3 sites.

Figure S3. Analysis of Wilson 2017 [23] gene expression data for GlyR1 and GlyR3 deletion strains of *C. thermocellum* DSM 1313.

Tables

Table S1. Position Specific Scoring Matrix of CcpA binding motif in *B. subtilis.*

Table S2 - Strains and plasmids.

Table S3 - Primers and probes.

Figure S1. Receiver Operator Curve for PSSM scored CcpA binding sites in *B. subtilis*.

The ROC curve quantifying the ability of various PSSM scores to identify CcpA binding sites in the *B. subtilis* genome. The 14 symbols in the figure represent PSSM scores ranging from 20 to 7 from left to right. The vertical arrow indicates a 1/10 true to false positive ratio occurring at a PSSM score of 12. The x-axis of the plot is scaled to emphasize the relevant portion of the ROC curve characterized by high PSSM scores.

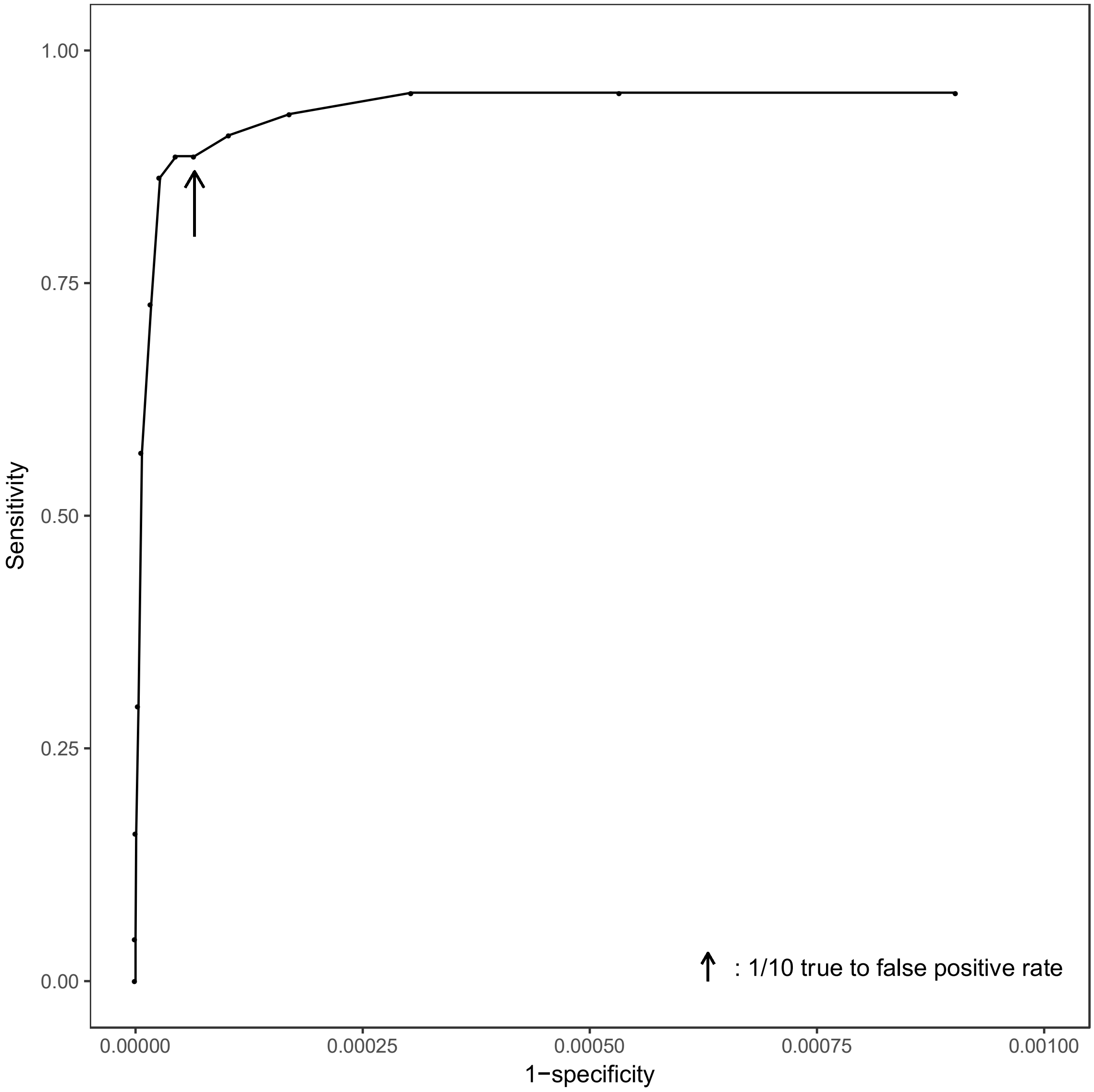

Figure S2. EMSA controls for GlyR1 binding to *celC* and *manB* GlyR3 sites.
Data indicate that GlyR1 does not bind to the GlyR3 sites of *celC* and *manB*.

celC*:** Lane1: *celC* DNA only (0.2 ng (0.156 nM)); Lane2: *celC* + GlyR1 (60 ng (152nM)); Lane3: *celC* + GlyR1 (150 ng (380nM). ***manB: Lane1: *manB* DNA only (0.2 ng (0.187 nM)); Lane2: *manB* + GlyR1 (60 ng (152nM)); Lane3: *manB* + GlyR1 (150 ng (380nM).

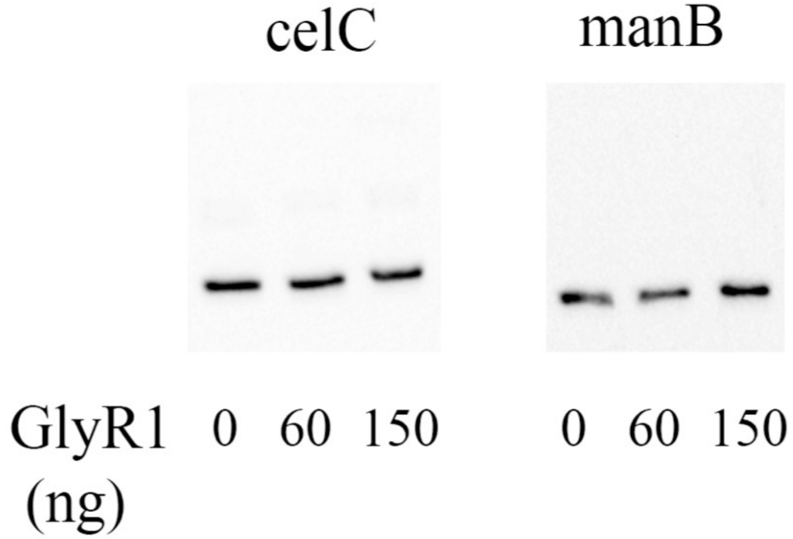

Figure S3. Analysis of Wilson 2017 [REF] gene expression data for GlyR1 and GlyR3 deletion strains of *C. thermocellum* DSM 1313. Data show that that GlyR1 is not involved in the regulation of these genes. The GlyR3 deletion strain shows increased expression of *CelC* and *licA*, but unchanged expression of *manB* and *celT*.

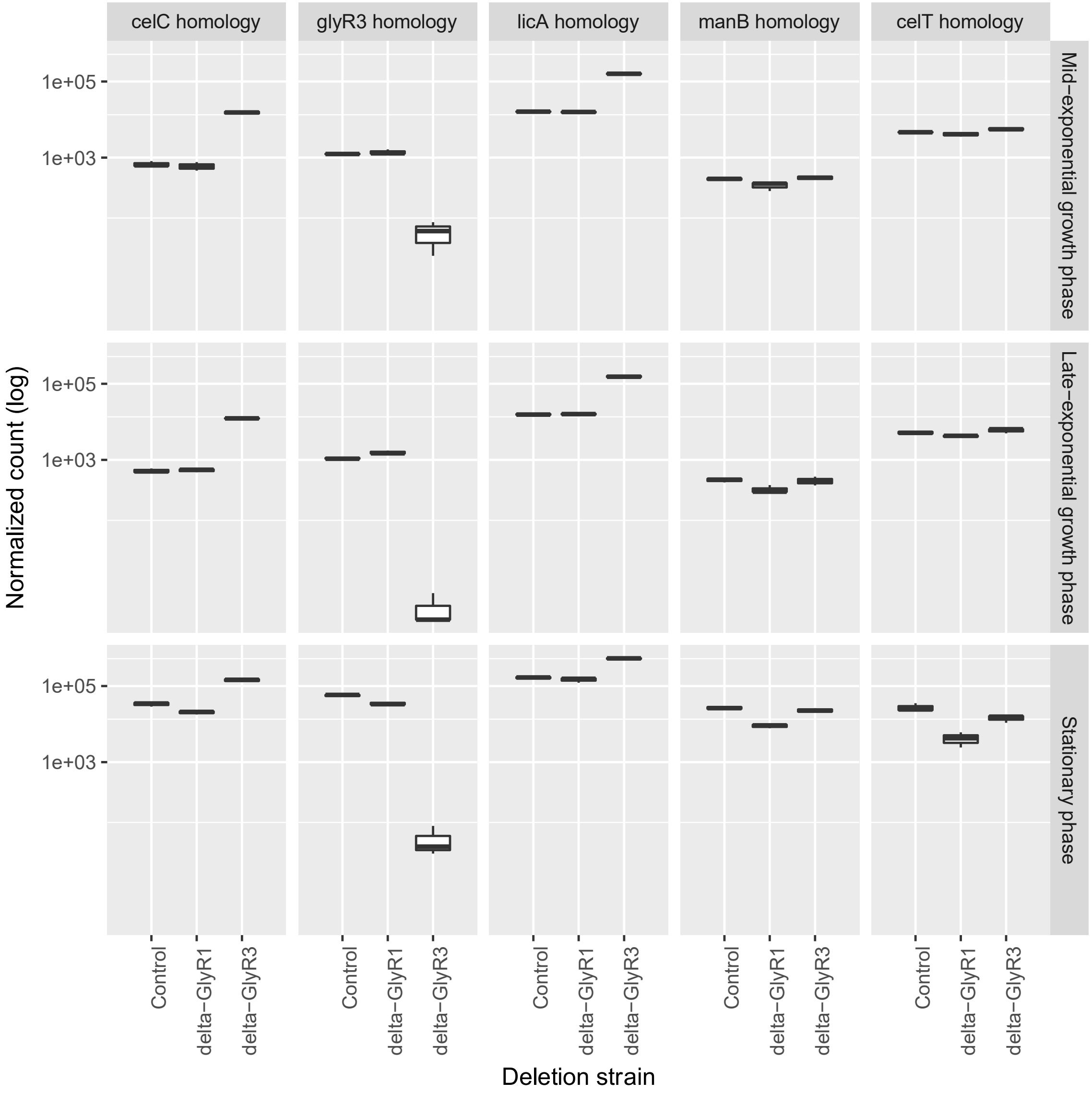

Table S2. Position Specific Scoring Matrix of CcpA binding motif in *B. subtilis.*

Base	Position													
	1	2	3	4	5	6	7	8	9	10	11	12	13	14
A	-1.3	-3.62	1.24	1.84	1.87	0.19	-2.62	-2.62	-1.62	-0.45	0.19	0.38	-0.81	1.47
G	-2.04	1.84	-1.3	-3.62	-3.62	1.13	-2.62	1.84	-0.62	-3.62	-1.3	-1.62	-3.62	-2.62
C	-3.62	-3.62	-2.62	-2.62	-3.62	-1.62	1.81	-3.62	0.63	-3.62	-2.04	-1.62	1.51	-2.04
T	1.67	-2.62	-0.04	-3.62	-3.62	-2.04	-3.62	-3.62	0.47	1.59	1.08	0.96	-1.3	-0.45
	T	G	A	A	A	G	C	G	C	T	T	T	C	A

Table S2 - Strains and plasmids. List of strains and plasmids used in this work.

Strains	Description	Reference or source
Clostridium thermocellum		
ATCC 27405	Wild type	ATCC 27405
Escherichia coli		
TOP10	*F- mcrA Δ(mrr-hsdRMS-mcrBC) φ80lacZΔM15 ΔlacX74 nupG recA1 araD139 Δ(ara-leu)7697 galE15 galK16 rpsL(Str^R) endA1 λ^-*	[16]
C2566	*fhuA2 lacZ::T7 gene1 [lon] ompT gal sulA11 R(mcr-73::miniTn10--Tet^S)2 [dcm] R(zgb-210::Tn10--Tet^S) endA1 Δ(mcrC-mrr)114::IS10*	This work
Plasmids		
pTXB1-glyR3	*glyR3* cloned into pTXB1	[16]
pCR2.1-TOPO		This work

Table S3 - Primers and probes. Primer and probe sequences used.

No	Sequence	Reference
1	F:glyR3-F-EcoRV-GCGCGATATCACCAGTGAAGAAATAGCAAAATTA	[16]
2	R:glyR3-R-Xhol- GCGCCTCGAGGAATTCCAAAGCCCTCTTGGTT	[16]
3	celCemsa-F-Biotin-CCGAATAAAAACTGGACAGAG	[16]
4	celCemsa-R-Unlab TCCTCCTGAAATATTGTGTTTTA	[16]
5	manBemsa-F-Biotin-TCGGTGAATGTTGAGGTTGA	This work
6	manBemsa-R-Unlab-TCCTGCTGAAATCTCTCTCCA	This work
7	celTemsa-F-Biotin-TGAGCAAATCAATTGTAATATGAAGA	This work
8	celTemsa-R-Unlab-TGCAGCAGAACGTTTCTTTC	This work
9	ControlF-Biotin-TGTTATCTTCGGTTAGCTCATCA	This work
10	ControlR-Unlab-TGGGAGGATGATACTGCTGTT	This work
11	celC-compt-F- AATGAACGCGCGTACATT	[16]
12	celC-compt-R- AATGTACGCGCGTTCATT	[16]
13	manB-compt-F- AATGTAAACGGTGTCAAT	This work
14	manB-compt-R- ATTGACACCGTTTACATT	This work
15	celT-compt-F- ATGTAAATCGGTTGCAGT	This work
16	celT-compt-F- ACTGCAACCGATTTACAT	This work
17	celCq-F-CGGGAACATATTGCCTTTGAAC	[16]
18	celCq-R-GGTGGAATCAATTTCCCTGATTG	[16]
19	Cthe_2811_F AACACATCCTTTCGGGTCAG	This work
20	Cthe_2811_R CACATCAAACCGTTCCCTCT	This work
21	Cthe_2812_F CTACAGCAGCAATTCGGTCA	This work
22	Chte_2812_R CATTGAAGAATCCCCGAAGA	This work
23	InvtCelC_F CCGAATAAAAACTGGACAGAAG	This work
24	InvtCelC_R CCAGTGGGCTTTCTGATGC	This work
25	InvtManB_F CGGCCAAATATGCCATAGAC	This work
26	InvtManB_R TCCTGAACCTGCTTGAGCTT	This work
27	InvtPCR_F CCATAAAACCGCCCAGTCTA	This work
